# Editorial: Treatment of brain metastases from non-small cell lung cancer: preclinical, clinical, and translational research

**DOI:** 10.3389/fonc.2025.1598159

**Published:** 2025-04-04

**Authors:** Jia Liu, Pengfei Zhou, Lei Deng, Jianxin Xue, Lin Zhou

**Affiliations:** ^1^ Department of Oncology, Chengdu First People’s Hospital, Chengdu, Sichuan, China; ^2^ Department of Thoracic Oncology, Meishan Cancer Hospital, Meishan, Sichuan, China; ^3^ Roswell Park Comprehensive Cancer Center, University at Buffalo, Buffalo, NY, United States; ^4^ Division of Thoracic Tumor Multimodality Treatment, Cancer Center and State Key Laboratory of Biotherapy, West China Hospital, Sichuan University, Chengdu, Sichuan, China

**Keywords:** brain metastases, non-small cell lung cancer, prognosis, treatment, genetic mutation detection

Non-small cell lung cancer (NSCLC) is the most common malignancy worldwide, with up to 50% of patients developing brain metastases (BMs) in their disease course and 70% of BMs developing multiple lesions (1). BMs have a significant negative impact on both survival and quality of life for patients with NSCLC. The Research Topic, which included 15 articles, aims to present new evidence for preclinical, translational, and clinical studies in NSCLC with BMs.

Sampat et al. comprehensively reviewed BMs in NSCLC, focusing on epidemiology, diagnosis, treatment strategies, and prognosis. Generally, local treatments, including surgery/stereotactic radiosurgery (SRS)/stereotactic radiotherapy (SRT), are effective for limited BMs, and whole-brain radiotherapy (WBRT) is used for more extensive disease. For patients with driver gene positive, BMs have shown excellent responses to small molecule tyrosine kinase inhibitors (TKIs). Furthermore, immunotherapies, such as checkpoint inhibitors, also demonstrate some effectiveness.

The pathological subtypes of NSCLC may be correlated with the occurrence and prognosis of BMs. Zhou et al. investigated the prognostic characteristics of BMs originating from invasive lung adenocarcinomas of distinct pathological subtypes. Clinical data from 156 patients were collected and analyzed. Patients were classified into two groups on the basis of pathological subtype: moderately to highly differentiated and poorly differentiated. The median overall survival (mOS) for the poorly differentiated group was 14.67 months (95% CI: 11.80–17.53 months), whereas it was 25.00 months (95% CI: 19.55–30.45 months) for the moderately to highly differentiated group (hazard ratio [HR] of 1.55, 95% CI: 1.06–2.25; *p* = 0.023). However, this study did not provide data on driver gene mutations or programmed death-ligand 1 (PD-L1) expression, which are established prognostic factors for NSCLC with BMs, and the prevalence of gene mutations and PD-L1 may vary across different pathological subtypes and influence the therapeutic effect (2–4). Wu et al. reported that pathological subtypes, together with sex, leukocyte count, and fibrinogen stage, were independent risk factors for predicting BMs in lung cancer patients. However, this study also did not provide data on driver gene mutations, which have been reported as risk factors for BMs in NSCLC (5).

TKIs serve as the cornerstone of treatment for driver gene-positive, such as epidermal growth factor receptor (EGFR)-mutant and anaplastic lymphoma kinase (ALK) rearrangement, NSCLC with BMs. In particular, new-generation TKIs exhibit high blood–brain barrier permeability and demonstrate superior efficacy against BMs (6, 7). As a result, brain metastases are no longer a critical prognostic factor for this patient population. Li et al. compared the prognoses of synchronous and metachronous BMs from NSCLC and reported that synchronous BMs (HR: 1.335, 95% CI: 1.076–1.657, *p* = 0.009), squamous carcinoma (HR: 1.361, 95% CI: 1.018–1.820, *p* = 0.037), and KPS score < 80 (HR: 1.392, 95% CI: 1.124–1.724, *p* = 0.002) were risk factors for OS. Interestingly, for patients with EGFR mutations, there was no significant difference in OS between synchronous and metachronous BMs (*p* = 0.270), which might indicate that the timing of BMs does not affect the prognosis of patients with EGFR mutations.

There have been numerous studies on the application of radiomics in NSCLC patients with BMs. Radiomics models based on multiparametric MRI can effectively predict the primary origin of brain metastases, clinical benefit, and progression-free survival (PFS) of immunotherapy for NSCLC patients with BMs (8, 9), and bimodal radiomics with CT and MRI (combining primary and BMs) achieves 86.7% accuracy in predicting the EGFR status (10). Similarly, Jiang et al. used CT perfusion imaging to predict pathological types of NSCLC with BMs, and reported that adenocarcinoma and squamous carcinoma patients presented significant differences in cerebral blood flow (CBF) and mean transit time (MTT) (p < 0.001, *p* = 0.012). For patients with difficulty to obtain pathological specimens and who are diagnosed with lung cancer clinically, it can assist in determining the pathological type. Xu et al. developed an MRI-based nomogram to predict immunotherapy effectiveness for NSCLC patients with BMs. The nomogram integrated intratumoral and peritumoral radiomic and clinical features and showed high accuracy in predicting the intracranial response and PFS.

Circulating tumor DNA (ctDNA) in cerebrospinal fluid (CSF) is more sensitive in reflecting the genetic features of BMs, especially in patients with leptomeningeal metastasis (LM) (11, 12). Similarly, Liu et al. investigated the utility of CSF and plasma ctDNA in detecting genetic mutations, including LM and brain parenchyma metastases (BPM) in NSCLC patients. The results revealed that CSF ctDNA had greater sensitivity than did plasma ctDNA for detecting mutations in LM patients, with all CSF ctDNA samples testing positive compared with 46.4% positivity in plasma ctDNA. In contrast, CSF ctDNA tests were negative in BPM patients. CSF ctDNA detected by next-generation sequencing can reflect the molecular characteristics and heterogeneity of NSCLC with LM, aiding in early LM detection. Furthermore, Abdulhaleem et al. explored the use of comprehensive genomic profiling via ctDNA to identify biomarkers for predicting clinical outcomes in NSCLC patients with BMs, and identified 72 genes significantly associated with clinical outcomes, with 14 genes linked to unfavorable outcomes and 36 to favorable outcomes.

It has been reported that peritumoral brain edema is correlated with prognosis, with extensive or prolonged edema duration generally associated with poorer outcomes (13, 14). Arrieta et al. investigated the predictive value of perilesional edema diameter (PED) associated with BMs for radiotherapy response in NSCLC patients, and reported that minor PED was independently associated with a better intracranial objective response rate (78.8% vs. 50%, OR 3.71, *p* = 0.018), longer median iPFS (11.8 vs. 6.9 months, HR 2.9, *p* < 0.001), and longer median OS (18.4 vs. 7.9 months, HR 2.1, *p* = 0.001). Cerebral radiation necrosis (CRN) is a relatively common late toxicity that severely influences patients’ quality of life following radiotherapy for BMs. The incidence rate of CRN after brain SRT/SRS for BMs has been reported to be 6.3–34% (15–17). Several studies have indicated that bevacizumab alleviates inflammatory responses and clinical symptoms in patients with CRN by inhibiting vascular endothelial growth factor (VEGF), thereby reducing vascular permeability and cerebral edema (18, 19). Zhang et al. explored whether bevacizumab could prevent CRN in NSCLC patients with BMs undergoing SRT, and reported that bevacizumab significantly reduced the incidence of CRN and/or symptomatic edema before (*p* = 0.036) and after (*p* = 0.015) inverse probability of treatment weighting (IPTW) adjustment.

The phase III FLAURA2 trial demonstrated that, compared with first-line osimertinib monotherapy, first-line osimertinib plus chemotherapy improved PFS (HR=0.62, 95% CI 0.49–0.79, *p* < 0.001) and CNS-PFS (HR=0.58, 95% CI 0.33–1.01) (20, 21). Chen et al. performed a meta-analysis to compare the efficacy and safety of combining chemotherapy with EGFR-TKIs (ETC) versus EGFR-TKIs monotherapy for EGFR-mutated NSCLC patients with BMs; the meta-analysis included seven studies based on five randomized clinical trials with 550 patients, and revealed that the ETC group had better OS (HR: 0.64 [0.48, 0.87]), PFS (HR: 0.42 [0.34, 0.52]), and central nervous system PFS (HR: 0.42 [0.31, 0.57]). However, the patients in the ETC group experienced more grade 3-5/serious adverse events. The previous meta-analysis suggested that immunotherapy combined with chemotherapy yielded a better objective response rate (ORR), PFS, and OS than immunotherapy alone for PD-L1-negative and driver-gene-negative nonsquamous NSCLC (22). Brown et al. assessed first-line chemoimmunotherapy and immunotherapy in NSCLC patients with BMs via data from the Australian Registry and biObank of Thoracic Cancers (AURORA), and reported that chemoimmunotherapy was associated with an improved intracranial objective response rate (iORR) (58% vs. 31%, *p*=0.01) and longer OS (HR 0.35; 95% CI 0.14–0.86, *p*=0.01), even quite more patients with PD-L1 > 50% in the immunotherapy alone group (95% vs. 27%).

The Research Topic contains three case reports. Xie et al. presented a case report of a 67-year-old male with pulmonary giant cell carcinoma, which is a rare subtype of NSCLC, and BMs were treated with penpulimab and anlotinib combined with cranial radiotherapy. The patient showed a significant reduction in both lung and brain lesions. Zhong et al. presented a case report of a 52-year-old female with EGFR-mutated NSCLC with LM. After treatment with high-dose aumolertinib (165 mg/day) and intrathecal pemetrexed via the Ommaya reservoir, the patient achieved significant remission, with LM progression-free survival exceeding 20 months. Chen et al. presented a case report of the efficacy of high-dose furmonertinib in treating LM from NSCLC. A 48-year-old man with EGFR-mutated NSCLC experienced rapid neurological symptom relief and 6-month survival post-LM diagnosis after receiving 160 mg/day furmonertinib.

In conclusion, this Research Topic presents new evidence on treatment, prognosis, radiomics analysis, ctDNA detection, and treatment-related adverse event management for NSCLC patients with BMs. However, several areas require further investigation to gain deeper insights into the mechanisms of BMs development, enhance treatment efficacy, and prolong patient survival. These include the tumor microenvironment in BMs from NSCLC, the mechanisms behind varying incidence across pathological/driver gene subtypes, optimal radiotherapy modalities/timing, and therapies for refractory BMs/LM after multiline treatments, etc.
